# Influence of Solid Oral Dosage Form Characteristics on Swallowability, Visual Perception, and Handling in Older Adults

**DOI:** 10.3390/pharmaceutics15041315

**Published:** 2023-04-21

**Authors:** Henriette Hummler, Susanne Page, Cordula Stillhart, Lisa Meilicke, Michael Grimm, Marwan Mannaa, Maik Gollasch, Werner Weitschies

**Affiliations:** 1 Pharma Technical Development, F. Hoffmann-La Roche Ltd., Grenzacher Str. 124, CH-4070 Basel, Switzerland; 2 Center of Drug Absorption and Transport, Department of Biopharmaceutics and Pharmaceutical Technology, Institute of Pharmacy, University of Greifswald, Felix-Hausdorff-Str. 3, 17487 Greifswald, Germany; 3 Department of Internal Medicine and Geriatrics, University Medicine Greifswald, Ferdinand-Sauerbruch-Straße, 17489 Greifswald, Germany

**Keywords:** solid oral dosage forms, swallowability, visual perception, handling, medicine acceptability, older adults, geriatric patients

## Abstract

Swallowability, visual perception, and any handling to be conducted prior to use are all influence factors on the acceptability of an oral dosage form by the patient. Knowing the dosage form preferences of older adults, as the major group of medication end users, is needed for patient-centric drug development. This study aimed at evaluating the ability of older adults to handle tablets as well as to assess the anticipated swallowability of tablets, capsules, and mini tablets based on visual perception. The randomized intervention study included 52 older adults (65 to 94 years) and 52 younger adults (19 to 36 years). Within the tested tablets, ranging from 125 mg up to 1000 mg in weight and being of different shapes, handling was not seen as the limiting factor for the decision on appropriate tablet size. However, the smallest sized tablets were rated worst. According to visual perception, the limit of acceptable tablet size was reached at around 250 mg for older adults. For younger adults, this limit was shifted to higher weights and was dependent on the tablet shape. Differences in anticipated swallowability with respect to tablet shapes were most pronounced for tablets of 500 mg and 750 mg in weight, independent of the age category. Capsules performed worse compared to tablets, while mini tablets appeared as a possible alternative dosage form to tablets of higher weight. Within the deglutition part of this study, swallowability capabilities of the same populations were assessed and have been reported previously. Comparing the present results with the swallowing capabilities of the same populations with respect to tablets, it shows adults’ clear self-underestimation of their ability to swallow tablets independent of their age.

## 1. Introduction

The oral route of administration is the most common. Solid oral dosage forms, SODFs, are the predominant type of dosage form for this route of administration. This also applies to older adults, being 65 years or older [1]. If tablets or capsules are too large, patients often modify their dosage forms with unknown consequences on the efficacy and/or safety. Swallowing difficulties are present in a large proportion of older adults and have been reported to be the main reasons for dosage form modifications [2,3,4]. Therefore, alternative dosage forms (e.g., mini tablets) have been developed in the past. These dosage forms have already been evaluated extensively in the context of pediatric medicines [5], however, assessments of alternative dosage forms in older adults are scarce [6].

Patients need to be willing to take their medication in order to adhere to their pharmacotherapy. Therefore, adequate acceptability is essential for the development of drug products [7]. The needs of the intended subset of patients have to be identified at the beginning of drug product development [7,8]. Drug product characteristics influencing the acceptability of medicines in older adults are stated in a reflection paper by the European Medicines Agency. Among others, appearance, swallowability, patient perception, and any handling to be conducted prior to use are listed [7].

Patient perception is described as the anticipated swallowability and palatability before the patient takes the medication. Furthermore, it is an appreciation of the color, size, shape, and viscosity of the individual product [7]. In the past, it has been reported that between 14% and 40% of the general population experience swallowing difficulties when taking medication [9,10,11,12]. These swallowing difficulties are often not due to any abnormalities found in a physical examination or laboratory findings such as videofluoroscopy and barium swallow studies, but are rather described as a psychogenic dysphagia, called phagophobia [13]. Younger age, female sex, as well as poor mental health were found to be associated with phagophobia [10,12,14]. Thus, phagophobia needs to be kept in mind when studying the patients’ visual perception of different dosage forms. Besides the size and shape of the different dosage forms, the color is expected to impact the visual perception. However, color was found to be of greater relevance for the identification and memorability of the products than for patients’ medication adherence [15].

The handling of dosage forms prior to use is part of medication management and is dependent on the type of dosage form. For tablets and capsules, handling includes the removal from the secondary and primary packaging as well as any necessary dose adjustment [7]. As multi-compartment compliant aids are commonly used by older adults, they need to pick the SODFs out of those boxes or from a table. Thus, in these cases, the need to remove packaging is eliminated. Impaired dexterity as well as a decrease in hand and grip strength are common within this patient population [16,17,18,19,20]. The aforementioned handling processes can become difficult for older adults, and thus might lead to non-adherence. It has been reported that tablets that are too small and too flat generally affect handling [21,22].

Swallowability, visual perception, as well as the handling of SODFs are all influenced by the size and shape of the individual dosage form. It is a balancing act to find the right size and shape of SODFs, allowing for acceptable handling and swallowability. This is especially true for the vulnerable patient population of older adults. Older adults, representing the greatest group of medication end users, are a heterogeneous group with diverse needs. Multimorbidity and consequently polypharmacy, being defined as an intake of five or more medications, are common within this patient population [23,24,25,26].

Thus, we aimed to gain information on acceptable sizes and shapes of SODFs for older adults with respect to swallowability, visual perception, and handling. Hereby, we focused on tablets, as they are the major type of SODFs being prescribed to older adults [1]. Since the results of the deglutition part of the study have already been reported [1], this article addresses the handling assessment of the different tablets as well as the visual perception assessment of different SODFs. Besides tablets, the visual perception part included capsules and mini tablets. Capsules are the second most common SODF and mini tablets have been proposed as an alternative dosage form to overcome swallowing difficulties. The acceptability of mini tablets has previously been tested in comparison with other alternative dosage forms, but a comparison to monolithic SODFs such as tablets is still missing [6]. Furthermore, the participants’ evaluation of swallowability after actual deglutition was compared to the one after the visual observation of SODFs. We also seeked to provide a limit to the upper and lower end of appropriate tablet size as well as appropriate tablet shapes for older adults. Additionally, we investigated the differences between older adults and a control group of younger adults.

## 2. Materials and Methods

### 2.1. Materials

**Figure 1:**
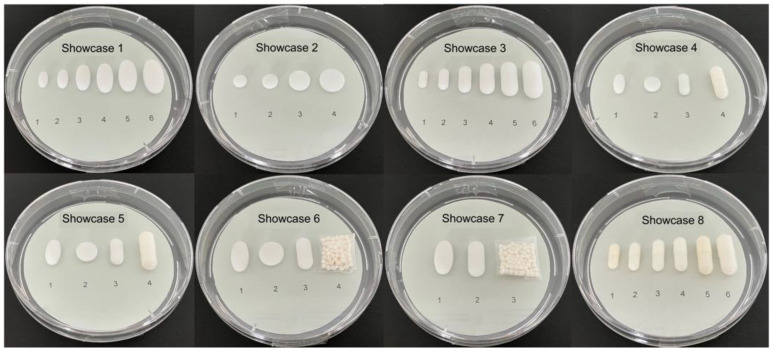
Eight different showcases were shown to the participants consecutively. Participants estimated the swallowability of the different dosage forms.

The tested dosage forms (Table 1) covered the most common weights, sizes, and shapes of SODFs prescribed to people 65 years and older in Germany [1]. Placebo tablets for the deglutition and handling part of the study were produced by direct compression followed by film coating using the same composition as previously reported [1]. Tablets for the visual perception part of the study were produced by 3D printing using selective laser sintering technology and were shown to the participants enclosed in showcases (Figure 1). Hydroxypropyl methylcellulose-based capsules were of white opaque color (Table 2). The uncoated placebo mini tablets were 2.3 mm in diameter and produced by direct compression.

The minimum cross sectional area of the dosage forms was calculated using the geometric dimensions.

**Table 1:** Description of tablet characteristics. The inclusion of the tablets in different parts of the study is specified under “deglutition”, “visual perception”, and “handling”. The group for the deglutition part refers to the experimental group assignment.

Shape	Weight [mg]	Diameter [mm]	Cap Radius [mm]	Thickness [mm]	Min. Cross Sectional Area [mm^2^]	Deglutition (Group)	Visual Perception	Handling
Round	125	7.40	9.13	2.97	18.21	-	x	x
Round	250	9.30	11.60	3.82	29.63	x (1)	x	x
Round	500	11.70	14.71	4.82	47.13	x (2)	x	x
Round	750	13.35	16.86	5.47	60.94	x (2)	x	x
**Shape**	**Weight [mg]**	**Length [mm]**	**Width [mm]**	**Thickness [mm]**	**Min. Cross Sectional Area [mm^2^]**	**Deglutition (Group)**	**Visual Perception**	**Handling**
Oval	125	9.50	4.95	3.30	13.89	-	x	x
Oval	250	12.00	6.25	4.24	22.17	x (2)	x	x
Oval	500	15.10	7.87	5.31	35.15	x (1)	x	x
Oval	750	17.30	9.02	6.12	46.12	x (1)	x	x
Oval	1000	19.00	9.90	6.66	55.64	x (2)	x	x
Oval	1250	20.48	10.67	7.24	64.59	-	x	-
Oblong	125	10.30	4.47	3.03	11.19	-	x	x
Oblong	250	12.75	5.53	3.81	17.11	-	x	x
Oblong	500	16.10	6.98	4.81	27.20	-	x	x
Oblong	750	18.50	8.02	5.48	35.96	-	x	x
Oblong	1000	20.50	8.89	6.07	44.24	x (1)	x	x
Oblong	1250	22.20	9.63	6.61	51.84	-	x	-

**Table 2:** Description of capsule characteristics. Anticipated weight was based on the weight of the capsule shells and a filling of the capsules with microcrystalline cellulose.

Capsule Size	Anticipated Weight [mg]	Length [mm]	Diameter [mm]	Min. Cross Sectional Area [mm^2^]
4	125	14.3	5.05	20.03
3	150	15.9	5.57	24.37
2	200	18.0	6.07	28.94
1	250	19.4	6.63	34.52
0	350	21.7	7.34	42.31
00	500	23.3	8.18	52.55

### 2.2. Handling Assessment

A total of 14 different tablets were placed on a table in front of the seated participant. Tablets were presented one after the other in a randomized order. The randomization was conducted using a Greco-Latin square design with two blocking factors, tablet shape and weight. Participants were asked to pick up each tablet from the table and to put it into a box situated approximately 30 cm above the table surface. Through this, the distance between table and mouth was mimicked [27]. Participants were allowed to use their hand of choice or to use both hands for help to handle the individual tablets. Participant-reported outcomes (PRO) and researcher reported outcomes (RRO) were used for the evaluation of the handling procedure. Participants were asked to rate the tablets as *not*, *moderately*, or *well to handle*. If tablets were *not* or only *moderately to handle*, participants were asked for the specific reasons. Possible answers were: tablet was too small, shape of the tablet impaired its handling, and/or tablet surface was too smooth. Any other reasons could also be given. Furthermore, the study team noted participants’ facial expression during handling. Additionally, the number of attempts and the time needed by participants to execute the task was recorded.

### 2.3. Visual Perception Assessment

To assess the visual perception, eight showcases including different SODFs were shown to the participants (Table 3 and Figure 1). Shape-showcases included tablets of the same shape but differing in weight as well as capsules of different sizes. However, the weight-showcases included SODFs of the same weight (e.g., tablets of different shape as well as a capsule or mini tablets). The showcases were presented in consecutive order. Participants were told to imagine swallowing the presented monolithic SODFs as if they were their own medications. For the mini tablets, they were told to imagine sprinkling them over soft food (i.e., yoghurt or apple sauce) and taking them with a spoon. For each of the showcases, the participants were asked a multiple choice question: *Which of the shown dosage forms do you think you are able to swallow?*

### 2.4. Study Population and Setting

Study population and setting have been reported previously [1]. The Ethics Committee of the University of Greifswald, Germany approved this study (internal registration number: BB 036/22). Participants were recruited between May and August 2022. Older participants were recruited at the geriatric ward of the local hospital in Wolgast, Germany, Altersmedizinisches Zentrum Kreiskrankenhaus Wolgast gGmbH. Younger participants were contacted via email and notices at the University of Greifswald, Germany.

Inclusion criteria were: participants’ age (≥65 years for the assessment in older adults; 18 to 64 years for the assessment in younger adults), signing informed consent, and a health status enabling participation in the study. The health status of older participants was assessed by physicians, psychologists, and a speech therapist at the Altersmedizinisches Zentrum Kreiskrankenhaus Wolgast gGmbH, Germany. Exclusion criteria were: diagnosed dementia as well as diagnosed dysphagia, cognitive impairments not allowing to give informed consent, and/or to follow study instructions (older participants needed a score of ≥25 in the Mini-Mental State Examination) [1].

The study implicated three study days for each participant. The visual perception and handling part of the study were conducted on the first and the third study day, respectively. The deglutition part was conducted repeatedly on each of the three study days [1]. Details of this part of the study have been described previously [1].

Demographic data of participants such as age, sex, body height and weight, any acute and chronic diseases, as well as the current medication were recorded. Furthermore, the participants were asked whether they managed their medication independently or if they were assisted. The participants were also asked whether they had encountered difficulties when swallowing drinks, foods, and/or medication in the present and/or past. For older participants, the results of the following assessments were recorded: Mini-Mental State Examination, Suhlman clock drawing test, and water swallowing test according to Daniels, if appropriate [28,29,30,31].

### 2.5. Statistical Analysis

The underlying sample size calculation for this study has been described previously in detail.

For the evaluation of the handling part, the McNemar test as well as the Bowker’s test were applied for paired data. The test application depended on the underlying k × k contingency tables. For analyzing the rating *well* vs. *not/moderately to handle*, k was two and therefore the Mc Nemar test was used. However, to analyze the rating of *well* vs. *moderately* vs. *not to handle*, k was three, and therefore Bowker’s test was applied. Evaluations were made separately for the two age categories. Tablets of the same shape and adjacent weight or of the same weight but different shape were compared to each other. The influence of age on the handling of the different tablets was studied using the rating of *well* vs. *not/moderately to handle* and applying Fisher’s exact test. For the evaluation of time needed to pick and move the different tablets, the values of mean ± standard deviation, minimum, and maximum are given separately for the two age categories.

The binary paired data of the visual perception part were compared using the McNemar test within the same age category. For the evaluation of same shaped tablets within a showcase, tablets with adjacent weights were compared. The same applied for capsules within showcase 8. However, within the weight-showcases, all dosage forms were compared to each other. To study the influence of age on the visual perception of dosage forms’ swallowability, Fisher’s exact test was used.

The swallowability rating after actual deglutition was compared to the rating after visual perception for each participant. For this comparison, the swallowability ratings (*swallowable* vs. *not swallowable*) previously reported were used [1]. From the visual perception part of the study, only data from the participants who actually swallowed each tablet were used. For the paired data, the McNemar test was applied.

For all tests, the *p*-values were corrected according to Benjamini and Hochberg, in order to control the false discovery rate and to account for multiple testing [32]. If applicable, the confidence interval (CI) boundaries were also adjusted according to Benjamini et al. [33].

*p*-values < 0.05 were deemed significant. Results of the Fisher’s exact test also included the odds ratios as well as the lower and upper CI boundaries. Data analysis was performed with JMP^®^ 15.2.0 (466311) (SAS Institute Inc., Cary, NC, USA).

## 3. Results

### 3.1. Study Population

**Figure 2:**
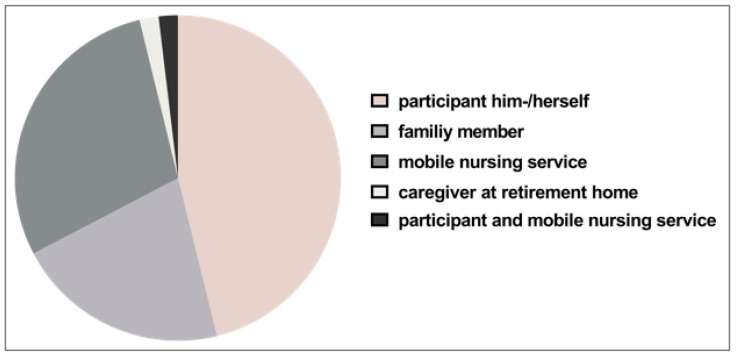
Responsibilities for medication management among the older participants.

The 52 older participants showed a mean age of 81.3 ± 7.3 years whereas the younger participants were on average 23.1 ± 3.8 years old. Women represented 67.3% of the older participants. Within the group of younger participants, the female share accounted for 55.8%. Among the older participants, all except one were diagnosed with at least two or more chronic conditions. Accordingly, polypharmacy applied to 96.2% of the older participants. While all the younger participants were themselves responsible for their medication management, more than half of older participants (53.8%) were assisted to some extent (Figure 2). We have reported previously the full details of participants’ characteristics [1].

### 3.2. Handling

#### 3.2.1. Rating

**Figure 3:**
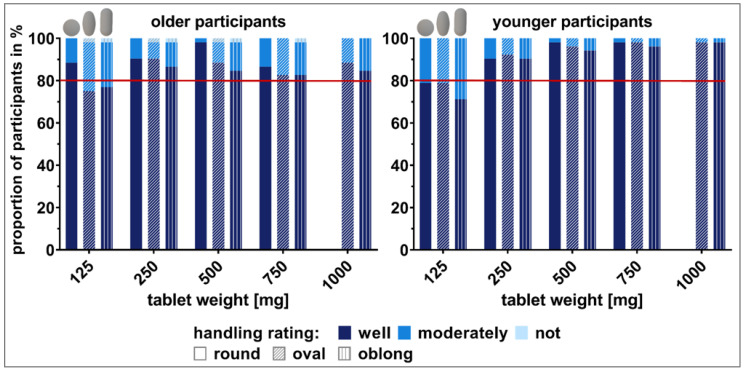
Handling of tablets differing in weight and shape rated as *not*, *moderately,* or *well to handle*. Data are shown separately for the older participants (**left** panel) and younger participants (**right** panel). Each participant gave a rating for every tablet. The value of 80% was used as a cutoff value for the data evaluation as it is a commonly used threshold for acceptability.

One older participant (1.9%) was not able to handle some of the tablets. All of the other participants, old and young, were able to handle all of the tablets with varying degrees of difficulty (Figure 3). We used 80% as a cutoff value for data evaluation as it is a commonly used threshold for acceptability [34]. Thus, all tablets exhibiting a weight of 250 mg or more were handled *well* by participants, independent of their age. Tablets of 125 mg were rated as *well to handle* in less than 80% by the older and younger participants, except for the round 125 mg tablet, which was rated *well to handle* by 88.5% of the older participants.

For the younger participants, there was a clear trend of increasing ease in handling with increasing tablet weight. Tablets weighing ≥500 mg were all comparable in terms of ease in handling. No such trend was seen in the older participants. For round tablets, a tablet weight of 500 mg was preferred for handling by older participants.

The Bowker’s test for tablet comparison in terms of handling based on the three-way split evaluation (*well* vs. *moderately* vs. *not to handle*) did not result in significant differences for any of the studied comparisons described in Section 2.5. Detailed test results are shown in the Supplementary Materials (Tables S1 and S2). The same held true when using the McNemar test to compare between *well* vs. *not/moderately to handle*. No significant difference was found for any of the comparisons, neither for older nor younger participants. However, for older participants, the comparisons between the oval 125 mg and the oval 250 mg, the oval 125 mg and the round 125 mg, the oblong 125 mg and the round 125 mg, as well as the round 500 mg and the oblong 500 mg tablet can be seen as borderto statistical significance (Supplementary Materials: Table S1).

In terms of age differences, all 750 mg tablets (oval, oblong, and round) as well as the oblong 1000 mg tablet were better to handle for the younger participants compared to their older counterparts and the comparisons were also borderto statistical significance. Detailed results of all of the described tests are shown in the Supplementary Materials (Table S3).

#### 3.2.2. Reasons for Handling Limitations

**Figure 4:**
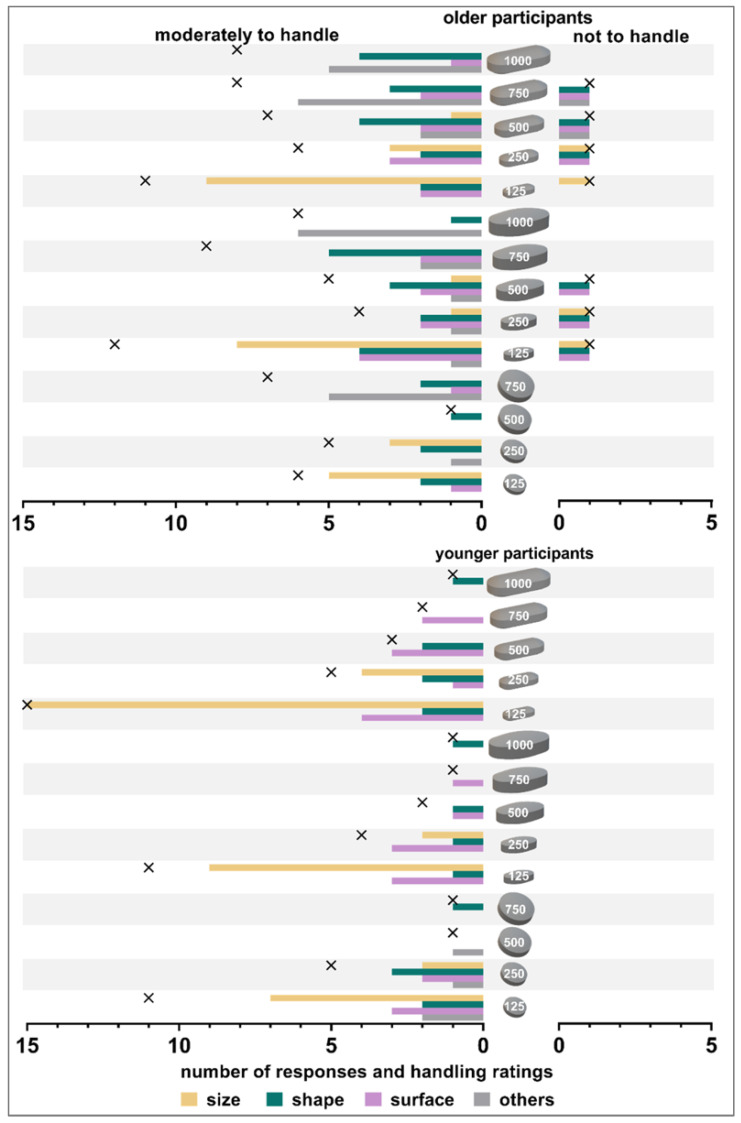
Number of responses and handling ratings (*moderately to handle* on the **left** panels and *not to handle* on the **right** panels). Crosses indicate the number of handling ratings (absolute numbers). Data are shown separately for the older participants (**top** panels) and younger participants (**bottom** panels).

The size of the tablets was the most frequent reason for reduced ability to handle tablets regardless of participants’ age. For tablets of 125 mg in weight, this implied too small a size while for tablets of 750 mg or 1000 mg weight, it implied a too big size (Figure 4). The latter only applied to older participants. One older participant stated that a bigger size required more finger force, thus impeding tablet’s handling. Older participants mentioned shape more often as debilitating for the handling of oval or oblongshaped tablets compared to round ones. Even if all tablets exhibited the same coating surface, some participants stated obstructive surface characteristics for different tablets. Other reasons for impaired handling of tablets stated by older participants were: surface is too rough, tablet is too big, and fear that tablet could slip away or that the tablet actually slipped away. Tablet being too big was stated most often. The younger participants mentioned a round shape, sticky surface, or too a flat shape as other reasons for impaired tablet handling.

#### 3.2.3. Characteristics Describing Handling

Compared to the younger participants, the older participants took more time to pick and move the different tablets (Table 4). Furthermore, the variability was higher for the older participants compared to the younger participants. Neither tablets’ weight nor shape influenced the time needed for handling.

Compared to the older participants, the younger participants needed less attempts to handle the different tablets (Table 5). This was also reflected in the time needed to handle the different tablets by the two age categories. Less than four percent of the younger participants needed more than one attempt to handle any of the tablets. In contrast, depending on the different tablets, between 7.7% and 25.0% of the older participants needed more than one attempt to handle the tablets. While younger participants needed a maximum of three attempts, some of the older participants needed up to five attempts depending on tablets’ characteristics.

The RRO of facial expression was not further used to assess tablet handling, as it was considered to be biased by the investigators’ personal perception and opinion.

### 3.3. Visual Perception

#### 3.3.1. Shape-Showcases

**Figure 5:**
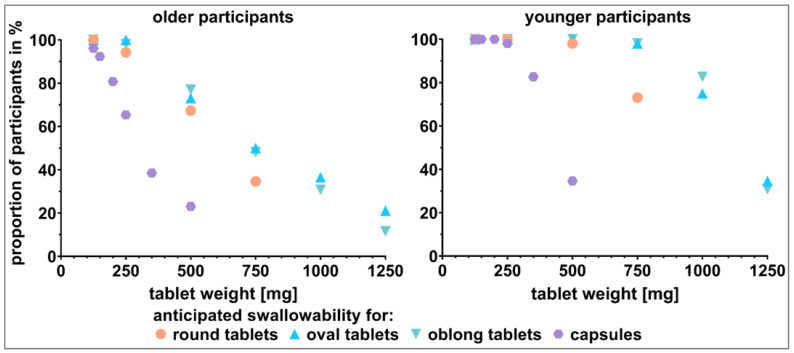
Visual perception of dosage forms’ swallowability by older participants (**left** panel) and younger participants (**right** panel). Capsules (size 4 to 00 with anticipated weight of 125 mg to 500 mg), round shaped tablets (125 mg to 750 mg), as well as oval and oblong shaped tablets (125 mg to 1250 mg) were shown to the participants in the shape-showcases.

The visual perception of the dosage forms’ swallowability was assessed by presenting different SODFs in showcases to the participants and asking them to estimate their ability to swallow the individual dosage forms. Only tablets exhibiting a weight of 250 mg or less were rated as *swallowable* by more than 80% of the older participants (Figure 5). The weight limit for the capsules was even lower. Capsule size 2 (anticipated weight of 200 mg) was already assumed *not* to be *swallowable* by 19.2% of older participants. With respect to length, capsule size 3 (anticipated weight of 150 mg) was of comparable size to the elongated shaped tablets of 500 mg. However, the estimated swallowability ratings of capsule sizes 2 and 1 were comparable to tablets exhibiting a weight of 500 mg. While the difference between tablets and capsules of 500 mg was evident (anticipated swallowability of around 70% and 23.1%, respectively), different tablet shapes did not seem to have an influence on the anticipated swallowability for tablets up to 500 mg in weight. The most pronounced difference between tablet shapes was observed for tablets of 750 mg in weight. The round shape performed worse compared to the oval and oblong shape. However, the estimated swallowability for all 750 mg tablets was distinctly below the 80% cutoff.

In contrast to the older participants, the limits for swallowable dosage forms was shifted to higher weights for younger participants (Figure 5). Again, taking 80% as the cutoff, the weight limit for tablets depended on their shape. For oblong shaped tablets, a tablet weight of 1000 mg was evaluated as *swallowable* by over 80% of the younger participants. The limit for oval tablets was reached at 750 mg. Over 20% of the younger participants judged round tablets exceeding a weight of 500 mg as *not swallowable*. For capsules, the limit was reached at an anticipated weight of 350 mg. As for the older participants, the most pronounced difference in tablet shapes was apparent for tablets of 750 mg in weight. The shape of 750 mg tablets was decisive for their classification as *swallowable* by over 80% of the younger participants.

#### 3.3.2. Weight-Showcases

**Figure 6:**
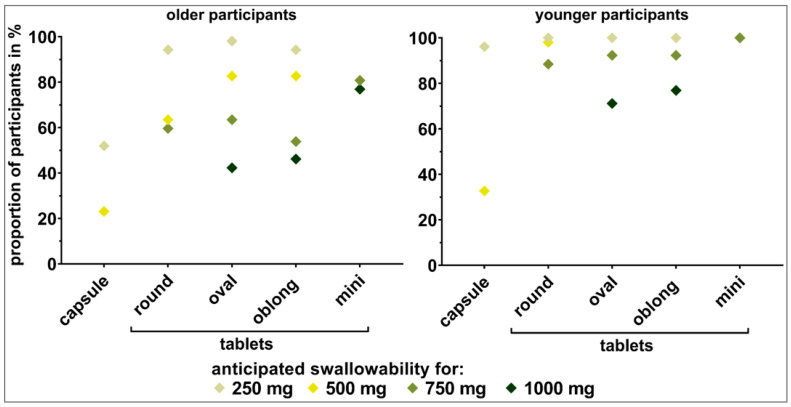
Visual perception of dosage forms’ swallowability by older participants (**left** panel) and younger participants (**right** panel). Capsules (250 mg and 500 mg), round shaped tablets (250 mg to 750 mg), oval and oblong shaped tablets (250 mg to 1000 mg), as well as mini tablets (750 mg and 1000 mg) were shown to the participants in showcases dependent on the weight of the dosage forms.

Capsules did not seem to be a suitable alternative to tablets for older participants when the SODFs were ≥250 mg in weight. While the oval and oblong tablets up to 500 mg in weight were considered as *swallowable* by >80% of the older participants, the same was true only up to 250 mg for the round tablets (Figure 6). For the older participants, differences in tablets’ shape were most pronounced for those of 500 mg in weight. Data from the shape-showcases showed the highest influence of shape for a tablet weight of 750 mg (Section 3.3.1). Worse swallowability was seen for the round shape (estimated swallowability < 80%) compared to the oval and oblong shape. For the 750 mg and 1000 mg weight classes, none of the monolithic tablets reached the 80% cutoff within the older participants. This is in line with the data from the shape-showcases. In contrast, mini tablets were deemed swallowable by around 80% of the older participants. Thus, mini tablets seem to be a promising alternative.

For the younger participants, the limits in terms of dosage form weight with respect to sufficient swallowability was shifted to higher ends. Within all four weight-showcases, the capsule of size 00 with 500 mg anticipated weight as well as the oval and oblong 1000 mg tablets were the only dosage forms not reaching the 80% cutoff. As for the older participants, the mini tablets seemed to be an alternative to monolithic tablets of 750 mg and 1000 mg in weight.

**Figure 7:**
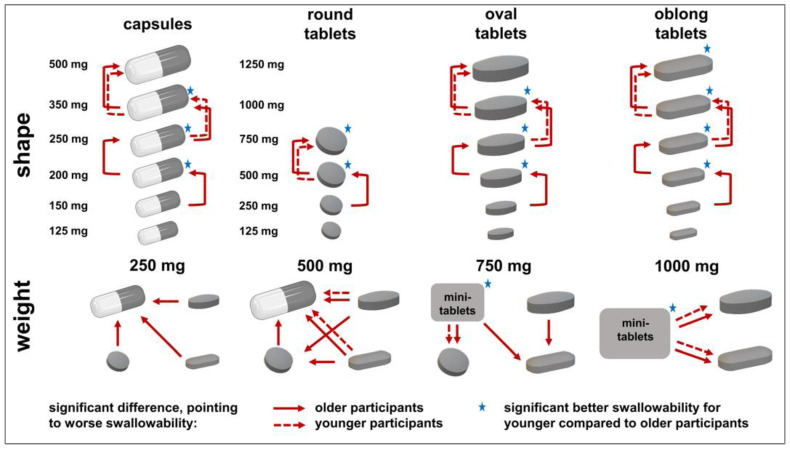
Significant differences according to the McNemar test for the data of visual perception. Arrows point to dosage forms showing a worse rating in terms of the anticipated swallowability. Results for the older participants (continuous line) and younger participants (dashed line) are shown. Comparisons between the dosage forms of the same kind were calculated for adjacent weights. For dosage forms of the same weight, all dosage forms were compared to each other. Stars indicate a significant difference between age categories according to Fisher’s exact test, implying better anticipated swallowability ratings for younger compared to older participants. To account for multiple testing, all calculated *p*-values were corrected according to Benjamini and Hochberg. *p*-values of <0.05 were deemed as significant.

Significant differences obtained by calculating the McNemar test for comparisons between dosage forms of the same kind or between those of the same weight and subsequent Benjamini–Hochberg correction supported observations made pursuant to Figure 5 and Figure 6 (Figure 7). For the older participants, significant difference occurred for tablets of the same shape when comparing 250 mg to 500 mg tablets or tablets of higher weight. For the younger participants, this only held true when comparing round shaped tablets with a weight of 500 mg and 750 mg. For tablets of elongated shape, comparisons starting at 750 mg to 1000 mg were of a significant nature. Figure 7 shows that a significant difference between age categories was to be expected for dosage forms of 500 mg or higher in weight. Detailed results of all of the described tests are shown in the Supplementary Materials (Tables S4–S6).

#### 3.3.3. Influence of Showcase Style on Visual Perception

**Figure 8:**
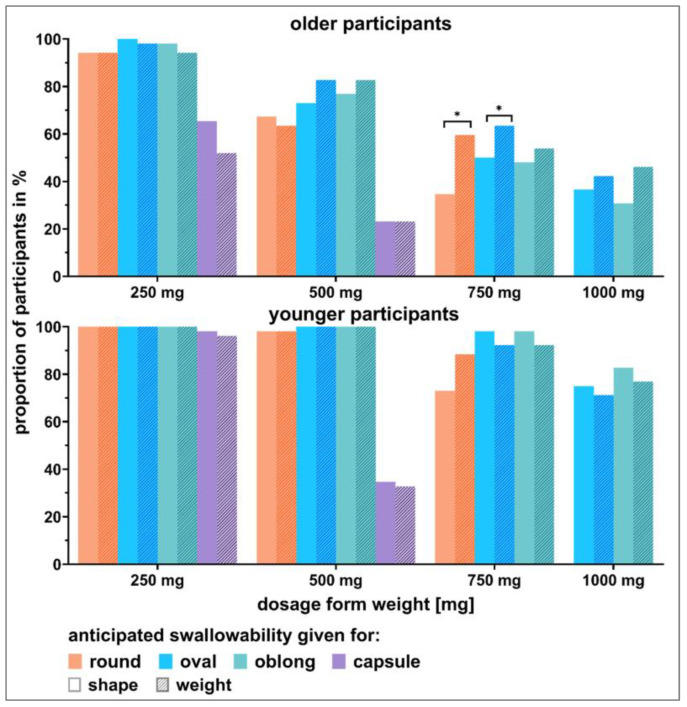
Influence of the showcase style (shape- or weight-showcase) on the visual perception of dosage forms’ swallowability. Data are shown for older participants (**upper** panel) as well as younger participants (**lower** panel). Significant differences according to the McNemar test and consequent Benjamini–Hochberg correction (*p*-value < 0.05) are marked with an asterisk.

Figure 8 shows that in some cases, the way of presenting dosage forms to participants changed their visual perception of the dosage forms’ swallowability, especially in older participants. In fact, older participants tended to rate dosage forms’ swallowability as worse if presented in the shape- compared to weight-showcases. For the round and oval shaped 750 mg tablets, this even led to a significant difference for the older participants. The 500 mg oval and oblong tablets were borderline *swallowable*, depending on the showcase (Figure 8; Supplementary Materials Tables S7 and S8).

#### 3.3.4. Influence of Sex on Visual Perception

**Figure 9:**
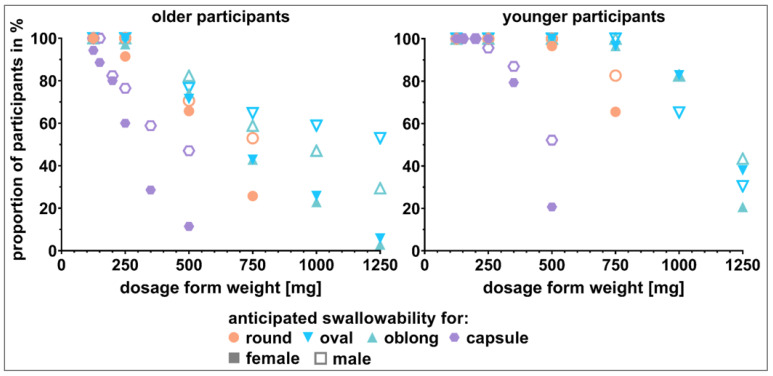
Influence of sex on the visual perception of the swallowability of the dosage forms presented within the shape-showcases. Data are shown separately for older participants (67.3% female; **left** panel) and younger participants (55.8% female; **right** panel).

**Figure 10:**
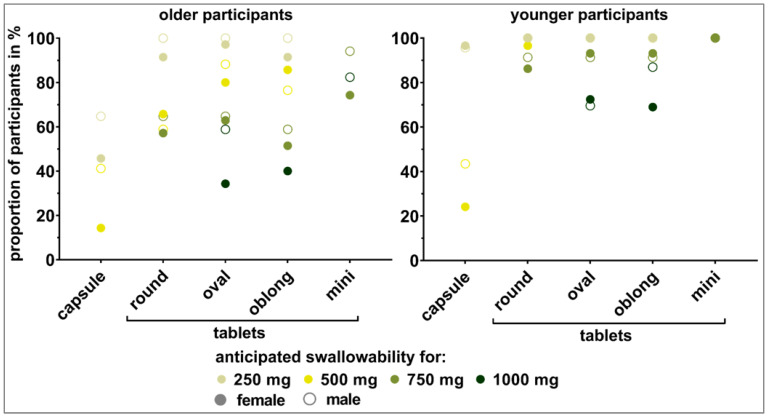
Influence of sex on the visual perception of the swallowability of the dosage forms presented within weight-showcases. Data are shown separately for older participants (67.3% female; **left** panel) and for younger participants (55.8% female; **right** panel).

As seen in Figure 9 and Figure 10, there was a trend of women rating the anticipated swallowability of SODFs worse compared to men, especially for larger sizes (>250 mg for capsules, >750 mg for tablets). An exception hereof were the oval shaped tablets within the group of younger participants, which were rated as worse by men than by women. Sex difference was more pronounced for older participants compared to their younger counterparts.

### 3.4. Visual Perception in Comparison to Actual Deglutition

**Figure 11:**
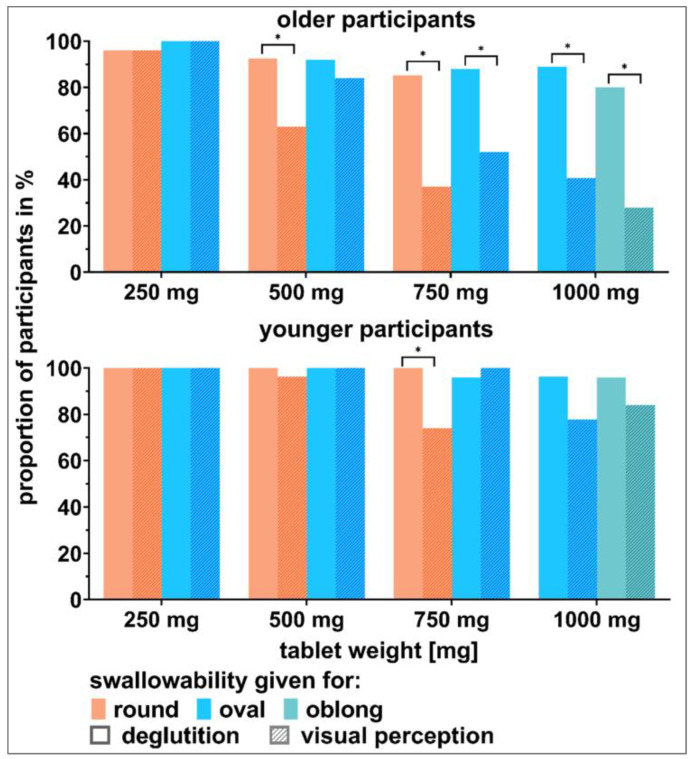
Comparison of the swallowability ratings after actual deglutition compared to those according to visual perception. Data of the visual perception only included ratings of those participants that had actually swallowed the particular tablets. Data are shown for older participants (**upper** panel) and younger participants (**lower** panel). Significant differences according to the McNemar test using paired data and consequent Benjamini–Hochberg correction (*p*-value < 0.05) are marked with an asterisk.

A comparison of the swallowability ratings after actual deglutition to those according to visual perception clearly showed participants’ self-underestimation (Figure 11). The older participants underestimated themselves to a higher extent compared to the younger participants. Applying blinding for the deglutition part clearly allowed to differentiate between participants’ actual and their estimated deglutition capabilities. Detailed results of all described tests are shown in the Supplementary Materials (Table S9).

## 4. Discussion

Older adults are not only the major group of medication end users, but are also a particularly heterogeneous and vulnerable patient population. However, information on the appropriate SODF size and shape for this patient population is still lacking [35]. Therefore, they were the focus group of this study. As the recruitment of older participants took place at the hospital, the majority of older participants was affected by two or more chronic conditions. This points to a rather vulnerable population and to their habit of regular medication intake [1].

The extent of handling (i.e., the sequent steps that need to be taken prior to medication administration), strongly depends on the type of dosage form. In our study population, more than half of the older participants (53.8%) received help in the medication management process from either family members, a mobile nursing service, or caregivers at retirement homes. However, the last step of medication handling before swallowing SODFs, namely, picking up a tablet from a blister, bottle, or a multi-compartment compliant aid and putting it into the mouth, is likely to be carried out by the patient. As the purpose of this study was to understand the influence of the shape and size of tablets on their handling, we coated all tablets with the same film-coating in order to exclude that differences in surface properties could affect the results. Surprisingly, no major difference was found between the ability to handle differently sized and shaped tablets when comparing the data of older vs. younger participants. Within the tested range of tablet sizes, the ability to handle a tablet did not influence the assessment of appropriate tablet sizes. Interestingly, the smallest tested tablets (125 mg tablet weight) were rated as the worst. Thus, smaller size was seen to complicate handling. Even if the smallest sized tablets were still able to be handled by the studied population, this might change when focusing on patients with more severe limitations in dexterity. Rheumatoid arthritis patients and, in general, female older patients were reported to have more difficulties with opening medication packaging [36]. Furthermore, Shariff et al. reported on small round tablets (<6 mm in diameter) being difficult to handle by older adults, as assessed with semi-structured interviews [21]. However, the older participants in this study were all able to handle the smallest tested round tablet of 7.4 mm in diameter and 88.5% even rated this tablet as *well to handle*. Round shaped tablets were rated better compared to the elongated tablets. This is in contrast to what we have seen for swallowability ratings in the deglutition part of the study [1], where a smaller minimum cross-sectional area was beneficial for swallowability. Younger participants seemed to be very critical in their ratings on handling. For the round and oblong shaped tablets of 125 mg, their ratings were even worse compared to those of the older participants. Furthermore, younger participants only needed a very short time and a low number of attempts for the handling of the different tablets. Even if the older participants needed more time and more attempts in the handling, their ratings were not distinctly worse compared to those of the younger participants. This might be an indication for more patience and higher frustration limits within the older age group due to their common confrontation with medication in their daily life. However, we expected distinctly worse ratings and more difficulties within older participants as impairment in manual and finger dexterity, loss of finger top feel, and decreased grip strength have been reported for this age group [18,20,37]. Using the ClinSearch Acceptability Score Test^®^, Vallet et al. described medication preparation and administration time of less than 20 s as short, between 21 s and 60 s as medium, and more than 60 s as long [38]. Adding up the time needed for handling and the time needed to swallow the different tablets that we reported previously [1], all intake attempts in this study can be described as short. Of note, our study did not include any removal from the secondary or primary packaging.

To detect the limit of tablet size within different tablet shapes in terms of the anticipated swallowability and to further test preferences in shape for same weight tablets, we used shape- as well as weight-showcases. The visual perception of dosage forms, thus the anticipated swallowability, is strongly influenced by the size, shape, and to some extent, the surface characteristics of the dosage form. When developing a new drug product, the estimated efficacious dose, the route of administration, and the type of dosage form influence the selection of the formulation technology. Thus, knowing the drug load, an estimation of dosage form size is possible. In order to develop patient-centric drug products that are accepted by patients, the size limits for the individual dosage forms need to be known. Thus, if an appropriate tablet size resulting in good swallowability is to be tested based on visual perception, one should pay attention to dosage forms’ weight being besides shape decisive for the final size of the SODFs. Weights of the dosage forms exhibiting the same largest dimension can be highly variable due to differences in density. This applies to the commonly used monolithic SODFs, namely, capsules compared to tablets.

In general, the results of the different studies assessing swallowability according to visual perception or retrospective experiences are difficult to compare as different methods have been used (Table 6). Furthermore, the study participants differed in their characteristics. The two studies conducted by Vallet et al. and Liu et al. resulted in different tablet size limits for people with swallowing difficulties and with dysphagia, respectively. Our visual perception data suggest that if an appropriate size for SODFs needs to be chosen for adults, the age and the general health status of the target patient population need to be respected. Comparing our data to the SODF sizes causing swallowing difficulties reported by Schiele et al., we observed lower limits for the older adults. In contrast, for younger adults, the size limits were shifted to a higher end. Earlier studies resulted in a general trend towards a higher acceptability of round shape for smaller sized tablets and elongated shapes for larger sized tablets [15,21]. This is in line with our results, which suggest that round tablets with a diameter of more than 9.30 mm (older adults) or 11.70 mm (younger adults) should be avoided, depending on the age of the patient population. Tablets with an elongated shape are more acceptable when targeting a weight of 250 mg or 500 mg.

**Table 6:** Studies assessing dosage form swallowability according to visual perception or retrospective experiences of the participants with their own medication.

Studied Population	Study Type	Tested Dosage Forms	Acceptable SODF Size ^1^	Reference
Older adults, mean age of 86 years, *n* = 938 tablet evaluations	ClinSearch Acceptability Test^®^	Retrospective analysis of patients’ regular medications, tablets ranged from 4.7–21.5 mm	Older adults w/o swallowing difficulties: tablets in general, older adults with swallowing disorders: only tablets < 6.5 mm	[39]
Older adults, mean age of 74 years, *n* = 156	Anticipated swallowability according to visual perception	Tablets: round and elongated shapes of 5–13 mm (d or l); hard gelatin capsules: size 4 to 00	Older adults with swallowing difficulties (11% of study population): tablet sizes < 11–13 mm (d or l) and capsules of size 0 or smaller	[6]
Adult patients, mean age of 61.8 ± 15.6 years, *n* = 1051	Patients reported swallowing difficulties among their own regular medications	Retrospective analysis of patients’ regular medications	Round tablets ≤ 8.1 mm (d) and ≤ 3.5 mm (h); oval tablets ≤ 13.2 mm (l), ≤6.6 mm (w), and ≤4.6 mm (h); oblong tablets ≤ 13.3 mm (l), ≤6.2 mm (w), and ≤4.9 mm (h) hard capsules: <6.4 mm (d), <17.5 mm (l); soft capsules: <8.0 mm (d), <18.3 mm (l)	[10]
Older adults: mean age of 79 years, *n* = 18; informal carers: mean age of 61 years, *n* = 7; health/social care professionals: *n* = 27	Semi-structured interviews	Tablets: round: 6–10 mm (d); oval: 12–16.5 mm (l), 7–8.9 mm (w); oblong: 18 mm (l), 7 mm (w)	Tablets > 10 mm (d) in case of a round shape should be provided in an elongated shape; good swallowability if tablets are not too thick	[21]
Adult patients, *n* = 278: 23–64 years, *n* = 53: ≥65 years	Anticipated swallowability according to visual perception	Tablets: flat or arched round: 8.1–15.1 mm (d), 3.1–5.5 mm (h); oblong: 12.2–22.2 mm (l), 6.3–8.9 mm (w), 4.4–7.2 mm (h); oval: 11.2–20.3 mm (l), 7.6–9.8 mm (w), 3.6–7.0 mm (h)	Small tablets: preference for strongly arched round shape; medium tablets: preference for oval shape; large tablets: preference for oblong, oblong curved, and oval shape	[15]
Older adults, mean age 81.3 ± 7.3 years, *n* = 52	Anticipated swallowability according to visual perception	Tablets: round: 7.40–13.35 mm (d); oval: 9.50–20.48 mm (l); oblong: 10.30–22.2 mm (l); capsules: size 4 to 00; mini tablets: 750–1000 mg	*Swallowable*: round tablets ≤ 9.30 mm (d); oval tablets ≤ 12.00 mm (l); oblong tablets ≤ 12.75 mm (l); capsules ≤ size 3 (≤15.9 mm (l)); mini tablets ≤ 1000 mg	Present study
Younger adults, mean age 23.1 ± 3.8 years, *n* = 52			*Swallowable*: round tablets ≤ 11.70 mm (d), oval tablets ≤ 17.3 mm (l), oblong tablets ≤ 20.5 mm (l) capsules ≤ size 0 (≤21.7 mm (l)) mini tablets ≤ 1000 mg	

^1^ Dosage forms’ dimensions are described in this table by d = diameter, l = length, w = width, and/or h = height.

The impact of different methods for the assessment of SODFs’ swallowability and their acceptability have been described previously [40]. This impact can also be seen when comparing the data of the deglutition part (i.e., the swallowing capabilities) to the data from the visual perception part (i.e., the anticipated swallowability). In addition, the influence of different study designs on the swallowability results became obvious when comparing the visual perception data of the shape- vs. weight-showcases within this study. The point of reference given to participants when evaluating the anticipated swallowability for different dosage forms seems to be relevant and influential. A few tablets were rated as swallowable by ≥80% of the participants or <80% depending on the type of showcase. This might indicate that these tablets represent the critical size for the individual tablet shape.

Phagophobia, an aversion to swallowing pills, has been described previously to be associated with female sex [12,14]. In this study, we saw a trend that women compared to men rated the dosage forms more often as *not swallowable* according to their visual perception. Previously, we have already reported on such a trend for younger adults according to their swallowability ratings after actual deglutition of different tablets [1]. This is in line with observations by Schiele et al. reporting on the association of increased prevalence for swallowing difficulties with medication and female sex [10]. Alternative dosage forms that would allow for better swallowability have gained much interest over the past years. Liu et al. reported on orodispersible tablets and mini tablets as the most accepted alternative dosage forms after effervescent tablets for older adults, but these dosage forms were not compared to conventional monolithic dosage forms such as capsules or tablets [6]. The results of the visual perception part of the present study suggest that mini tablets are a promising alternative to larger sized tablets of 750 mg and even higher in weight. Mini tablets might be considered for adults independent of their age. Nevertheless, a high dose strength might be restricted due to limited drug load per unit dosage form, and furthermore, the handling of such mini tablets might be difficult for older adults [35].

The comparison between the anticipated swallowability according to visual perception and the actual ability to swallow showed a great difference. In particular, participants 65 years or older largely underestimated their ability to swallow tablets. Even though the blinding used during the deglutition part of this study was far off a realistic intake scenario [1], it allowed for an interesting comparison between the visual perception, which is psychologically biased, and the participants’ actual ability to swallow [21]. The observed difference underlines the need and the potential of training as well as educational interventions to reduce and/or overcome psychological barriers in medication intake [10,14,41]. Independent of the method used to assess swallowability, either by visual perception or the actual deglutition of test objects, we saw limited swallowability for the older participants compared to the younger participants. However, in the past, difficulties in swallowing medication among adults has been associated with younger age and female sex [10]. Differences between the literature values and those in the study have been discussed previously [1].

Within the deglutition part that we reported previously, a stricter qualitative assessment of swallowability resulted in *well swallowable* tablets for older participants in case of oval shape and 250 mg tablet weight [1]. For the younger participants, both 250 mg tablets as well as the oval 500 mg tablet were considered as *well swallowable*. Given the good swallowability, ≥80% of participants independent of their age were willing to take them on a daily basis over one week or several months. Furthermore, these tablet sizes were considered swallowable following visual perception. Therefore, these tablet sizes and shapes can be seen as appropriate and would be well-suited to achieve a possibly high adherence of adult patients independent of their age. However, if high doses have to be administered, either dosage form size or the number of single units would need to be increased. The present study did not consider this aspect, but the pill number might be of interest for further investigations, especially when considering drug product development for older adults, who are often subject to polypharmacy.

Medication needs to be efficacious as well as easy and safe to use for the patient [42]. For the intake of SODFs, swallowability is important in terms of the ease of use as well as from a safety perspective. To be efficacious, medicines need to be taken as intended, and thus the medicines’ acceptability is of great importance. The SODFs’ size and shape influence the appearance, swallowability, patient perception, and any handling to be conducted prior to use [7]. In this study, we investigated the swallowability, visual perception, and handling, allowing for a clear comparison between these factors, as tablets of the same size and shape were tested. Thus, the results can help to find an appropriate size and shape for monolithic SODFs, allowing us to balance the different aspects of medicine acceptability. Even if there is no general agreement on the acceptability limits, we used that of 80%. However, these limits might differ and are likely to need adjustment depending on the target patient population.

## 5. Conclusions

The evaluation of the handling performance showed that handling was not the limiting factor for the decision on the appropriate tablet size and shape for the studied population. Data of the visual perception resulted in lower size limits for the older compared to younger participants. Mini tablets were found to be a possible alternative to monolithic SODFs of 750 mg or higher in weight independent of adults’ age. However, when comparing the anticipated swallowability for capsules and tables of the same weight, tablets were rated as better swallowable. The SODF size limits were distinctly lower according to visual perception compared to the results of the deglutition assessment. Great self-underestimation was observed independent of participants’ age, but was even more pronounced at a higher age.

## Figures and Tables

**Table 3:** Composition of the eight showcases representing different SODFs.

Showcase	Type of Showcase	Included Dosage Forms
1	shape	Oval shaped tablets— 125 mg, 250 mg, 500 mg, 750 mg, 1000 mg, 1250 mg
2	shape	Round, biconvex shaped tablets— 125 mg, 250 mg, 500 mg, 750 mg
3	shape	Oblong shaped tablets— 125 mg, 250 mg, 500 mg, 750 mg, 1000 mg, 1250 mg
4	weight	Dosage forms of 250 mg weight— oval, round, and oblong shaped tablets as well as one capsule (size 1)
5	weight	Dosage forms of 500 mg weight— oval, round, and oblong shaped tablets as well as one capsule (size 00)
6	weight	Dosage forms of 750 mg weight— oval, round, and oblong shaped tablets as well as mini tablets
7	weight	Dosage forms of 1000 mg weight— oval and oblong shaped tablets as well as mini tablets
8	shape	Capsules— sizes 4, 3, 2, 1, 0, 00

**Table 4:** Time needed to handle the different tablets. Data are given for older participants (**left**) and younger participants (**right**).

Tablet	Older Participants				Younger Participants			
	Mean [s]	StdDev [s]	Min [s]	Max [s]	Mean [s]	StdDev [s]	Min [s]	Max [s]
125 mg round	3.0	1.0	2	6	1.1	0.3	1	3
250 mg round	2.9	0.7	2	5	1.0	0.0	1	1
500 mg round	2.7	0.6	2	4	1.0	0.0	1	1
750 mg round	3.1	1.1	2	8	1.0	0.0	1	1
125 mg oval	3.2	1.5	2	12	1.0	0.2	1	2
250 mg oval	3.2	1.6	2	13	1.0	0.0	1	1
500 mg oval	3.2	1.6	2	10	1.0	0.0	1	1
750 mg oval	3.1	1.2	2	8	1.0	0.0	1	1
1000 mg oval	3.0	0.9	2	5	1.0	0.0	1	1
125 mg oblong	3.3	2.0	2	16	1.1	0.2	1	2
250 mg oblong	3.3	1.9	2	14	1.0	0.1	1	2
500 mg oblong	3.2	1.6	2	13	1.0	0.0	1	1
750 mg oblong	3.2	1.8	2	14	1.0	0.0	1	1
1000 mg oblong	2.9	0.8	2	5	1.0	0.1	1	2

**Table 5:** Number of attempts needed by the participants to pick up the different tablets from a table and to put them into a box 30 cm above the table surface. Data are shown separately for older participants (**left**) and younger participants (**right**).

	Older Participants						Younger Participants					
Tablet	Number of Attempts [%]					Not to Be Handled [%]	Number of Attempts [%]					Not to Be Handled [%]
	1	2	3	4	5		1	2	3	4	5	
125 mg round	88.5	3.8	7.7	0.0	0.0	0.0	96.2	1.9	1.9	0.0	0.0	0.0
250 mg round	92.3	5.8	1.9	0.0	0.0	0.0	100.0	0.0	0.0	0.0	0.0	0.0
500 mg round	92.3	7.7	0.0	0.0	0.0	0.0	100.0	0.0	0.0	0.0	0.0	0.0
750 mg round	86.5	7.7	1.9	1.9	1.9	0.0	98.1	1.9	0.0	0.0	0.0	0.0
125 mg oval	75.0	23.1	0.0	0.0	0.0	1.9	96.2	1.9	1.9	0.0	0.0	0.0
250 mg oval	78.8	19.2	0.0	0.0	0.0	1.9	98.1	1.9	0.0	0.0	0.0	0.0
500 mg oval	88.5	7.7	0.0	0.0	1.9	1.9	98.1	1.9	0.0	0.0	0.0	0.0
750 mg oval	82.7	15.4	1.9	0.0	0.0	0.0	100.0	0.0	0.0	0.0	0.0	0.0
1000 mg oval	84.6	11.5	1.9	1.9	0.0	0.0	100.0	0.0	0.0	0.0	0.0	0.0
125 mg oblong	82.7	9.6	1.9	3.8	0.0	1.9	96.2	3.8	0.0	0.0	0.0	0.0
250 mg oblong	80.8	11.5	3.8	1.9	0.0	1.9	98.1	1.9	0.0	0.0	0.0	0.0
500 mg oblong	80.8	15.4	1.9	0.0	0.0	1.9	100.0	0.0	0.0	0.0	0.0	0.0
750 mg oblong	86.5	7.7	1.9	0.0	1.9	1.9	100.0	0.0	0.0	0.0	0.0	0.0
1000 mg oblong	90.4	3.8	5.8	0.0	0.0	0.0	100.0	0.0	0.0	0.0	0.0	0.0

## Supplementary Materials

The following supporting information can be downloaded at: https://www.mdpi.com/article/10.3390/pharmaceutics15041315/s1, Table S1: Results of McNemar and Bowker’s test for data of handling for older participants. In order to create 2 × 2 contingency tables for the McNemar test, *well to handle* was compared to *not/moderately to handle*. Given is the *p*-value for both tests.; Table S2: Results of McNemar and Bowker’s test for data of handling for younger participants. In order to create 2 × 2 contingency tables for the McNemar test, *well to handle* was compared to *not/moderately to handle*. Given is the *p*-value for both tests.; Table S3: Results of Fisher’s exact test for the comparison of the different tablets between the two age categories. Worse handling was anticipated for older participants. Given are the *p*-value, odds ratio, as well as the lower and upper 95% confidence interval boundaries.; Table S4: Results of McNemar test for data of visual perception for older participants. Given is the *p*-value together with the corrected *p*-value according to Benjamini and Hochberg.; Table S5: Results of McNemar test for data of visual perception for younger participants. Given is the *p*-value together with the corrected *p*-value according to Benjamini and Hochberg.; Table S6: Results of Fisher’s exact test for comparison of visual perception, data from older and younger participants. Given is the *p*-value, the odds ratio, as well as upper and lower 95% confidence interval boundaries. The given results always implicate a better swallowability rating by younger participants compared to older participants or the same rating for both age categories.; Table S7: Results of McNemar test to study the influence of visual display on visual perception of different dosage forms. Anticipated swallowability ratings of the same dosage form displayed in the shape- vs weight-showcase were compared. Data are shown for older participants. *p*-value for the individual comparisons are given.; Table S8: Results of McNemar test to study the influence of visual display on visual perception of different dosage forms. Anticipated swallowability ratings of the same dosage form displayed in the shape- vs weight-showcase were compared. Data are shown for younger participants. *p*-value for the individual comparisons are given.; Table S9: Results of McNemar test to study the difference in swallowability after actual deglutition compared to visual perception for the different tablets. Data of visual perception only includes ratings of those participants having actually swallowed the particular tablets. This allows to study significant differences in swallowability ratings for the individual participant. For all comparisons, worse swallowability ratings were anticipated for the visual perception part. Data are shown for older and younger participants. *p*-value for the individual comparisons are given.
