# Supplementary material for: Influence of Solid Oral Dosage Form Characteristics on Swallowability, Visual Perception, and Handling in Older Adults
Source: Pharmaceutics. 2023 Apr 21;15(4):1315. doi: 10.3390/pharmaceutics15041315 (PMC10142368; doi:10.3390/pharmaceutics15041315)
Supplement: Supplementary file 1 [file pharmaceutics-15-01315-s001.zip › pharmaceutics-2333832-supplementary.pdf]

Supplementary Materials: Influence of Solid Oral Dosage Form Characteristics on Swallowability, Visual Perception, and Handling in Older Adults

Henriette Hummler, Susanne Page, Cordula Stillhart, Lisa Meilicke, Michael Grimm, Marwan Mannaa, Maik Gollasch and Werner Weitschies

Supplementary Information:

Table S1: Results of McNemar and Bowker's test for data of handling for older participants. In order to create 2 x 2 contingency tables for the McNemar test, *well to handle* was compared to *not/moderately to handle*. Given is the *p*-value for both tests.

Showcase	Dosage forms (<, >, = indicating trend of handling rating)			McNemar		Bowker	
				p-value	Corrected *p*-value	*p*-value	Corrected *p*-value
**oval - shape**	**125 mg**	**<**	250 mg	0.0114	0.1049	0.0937	0.4498
	250 mg	>	500 mg	0.7389	0.7389	0.9905	0.9905
	500 mg	>	750 mg	0.3657	0.6705	0.8451	0.9905
	750 mg	<	1000 mg	0.3657	0.6705	0.8451	0.9905
round - shape	125 mg	<	250 mg	0.7389	0.7389	0.9905	0.9905
	250 mg	<	500 mg	0.0455	0.1430	0.2615	0.7845
	500 mg	>	750 mg	0.0339	0.1243	0.2123	0.7279
oblong - shape	125 mg	<	250 mg	0.0588	0.1617	0.3116	0.8309
	250 mg	>	500 mg	0.7389	0.7389	0.9905	0.9905
	500 mg	>	750 mg	0.6547	0.7389	0.9776	0.9905
	750 mg	<	1000 mg	0.5637	0.7295	0.8013	0.9905
125 mg - weight	oval	<	round	0.0196	0.1078	0.0919	0.4498
	oval	<	oblong	0.7389	0.7389	0.0919	0.4498
	round	>	oblong	0.0143	0.1049	0.0719	0.4498
250 mg - weight	oval	=	round	1	1	0.7530	0.9905
	oval	>	oblong	0.4795	0.7033	0.9189	0.9905
	round	>	oblong	0.5271	0.7248	0.7774	0.9905
500 mg - weight	oval	<	round	0.0253	0.1113	0.1718	0.6872
	oval	<	oblong	0.3173	0.6705	0.8013	0.9905
	round	>	oblong	0.0082	0.1049	0.0719	0.4498
750 mg - weight	oval	<	round	0.4795	0.7033	0.9189	0.9905
	oval	=	oblong	1	1	0.8013	0.9905
	round	>	oblong	0.4795	0.7033	0.7667	0.9905
1000 mg - weight	oval	>	oblong	0.3173	0.6705	0.8013	0.9905

Table S2: Results of McNemar and Bowker's test for data of handling for younger participants. In order to create 2 x 2 contingency tables for the McNemar test, *well to handle* was compared to *not/moderately to handle*. Given is the p-value for both tests.

Showcase	Dosage forms (<, >, = indicating trend of handling rating)			McNemar		Bowker	
				p-value	Corrected *p*-value	*p*-value	Corrected *p*-value
**oval - shape**	**125 mg**	**<**	250 mg	0.0348	0.2958	0.2164	0.9536
	250 mg	<	500 mg	0.4142	0.5637	0.881	0.9536
	500 mg	<	750 mg	0.3173	0.5637	0.8013	0.9536
	750 mg	=	1000 mg	1	1	1	1
round - shape	125 mg	<	250 mg	0.0578	0.3275	0.308	0.9536
	250 mg	<	500 mg	0.1025	0.4356	0.4459	0.9536
	500 mg	=	750 mg	1	1	1	1
oblong - shape	125 mg	<	250 mg	0.0124	0.2108	0.1001	0.9536
	250 mg	<	500 mg	0.4142	0.5637	0.881	0.9536
	500 mg	<	750 mg	0.5637	0.5637	0.9536	0.9536
	750 mg	<	1000 mg	0.1573	0.5348	0.9536	0.9536
125 mg - weight	oval	=	round	1	1	1	1
	oval	>	oblong	1	1	1	1
	round	>	oblong	0.2482	0.5637	0.7212	0.9536
250 mg - weight	oval	>	round	0.5637	0.5637	0.9536	0.9536
	oval	>	oblong	0.5637	0.5348	0.9536	0.9536
	round	=	oblong	1	1	1	1
500 mg - weight	oval	<	round	0.5637	0.5637	0.9536	0.9536
	oval	>	oblong	0.5637	0.5637	0.9536	0.9536
	round	>	oblong	0.3173	0.5637	0.8013	0.9536
750 mg - weight	oval	=	round	1	1	1	1
	oval	>	oblong	0.5637	0.5637	0.9536	0.9536
	round	>	oblong	0.5637	0.5637	0.9536	0.9536
1000 mg - weight	oval	=	oblong	1	1	1	1

Table S3: Results of Fisher's exact test for the comparison of the different tablets between the two age categories. Worse handling was anticipated for older participants. Given are the *p*-value, odds ratio, as well as the lower and upper 95% confidence interval boundaries.

Compared tablet	*p*-value	Odds ratio	Lower 95% CI	Upper 95% CI	Corrected *p*-value
125 mg round	0.9451	0.49	0.17	1.43	0.9451
250 mg round	0.6294	1	0.27	3.68	0.8011
500 mg round	0.7524	1	0.06	16.42	0.8770
750 mg round	0.0299	7.93	0.94	66.98	0.1047
125 mg oval	0.4082	1.24	0.50	3.10	0.6350
250 mg oval	0.5	1.28	0.32	5.05	0.7000
500 mg oval	0.1347	3.26	0.63	16.97	0.2694
750 mg oval	0.0079	10.67	1.30	87.65	0.1047
1000 mg oval	0.0561	6.65	0.77	57.35	0.1571
125 mg oblong	0.8144	0.74	0.31	1.79	0.8770
250 mg oblong	0.3801	1.46	0.43	4.94	0.6350
500 mg oblong	0.1004	2.97	0.74	11.90	0.2343
750 mg oblong	0.0258	5.23	1.07	25.54	0.1047
1000 mg oblong	0.0156	9.27	1.12	77.07	0.1047

Table S4: Results of McNemar test for data of visual perception for older participants. Given is the *p*-value together with the corrected *p*-value according to Benjamini and Hochberg.

**Showcase**	**Dosage forms (<, >, = indicating trend of swallowability rating)**			***p*-value**	**Corrected *p*-value**
oval - shape	125 mg	=	250 mg	1	1
	250 mg	>	500 mg	0.0020	0.0040*
	500 mg	>	750 mg	0.0005	0.0018*
	750 mg	>	1000 mg	0.0082	0.0128*
	1000 mg	>	1250 mg	0.0047	0.0077*
round - shape	125 mg	>	250 mg	0.0833	0.1034
	250 mg	>	500 mg	0.002	0.0040*
	500 mg	>	750 mg	<0.0001	<0.001*
oblong - shape	125 mg	>	250 mg	0.3173	0.3173
	250 mg	>	500 mg	0.0009	0.0027*
	500 mg	>	750 mg	0.0001	<0.001*
	750 mg	>	1000 mg	0.0027	0.0051*
	1000 mg	>	1250 mg	0.0016	0.0040*
capsules	4	>	3	0.1573	0.1827
	3	>	2	0.0143	0.0206*
	2	>	1	0.0114	0.0171*
	1	>	0	0.002	0.0040*
	0	>	00	0.0047	0.0077*
250 mg - weight	oval	>	round	0.3173	0.3173
	oval	>	oblong	0.1573	0.1827
	oval	>	capsule	<0.0001	<0.001*
	round	=	oblong	1	1
	round	>	capsule	<0.0001	<0.001*
	oblong	>	capsule	<0.0001	<0.001*
500 mg - weight	oval	>	round	0.0016	0.004*
	oval	=	oblong	1	1
	oval	>	capsule	<0.0001	<0.001*
	round	<	oblong	0.0039	0.0070*
	round	>	capsule	<0.0001	<0.001*
	oblong	>	capsule	<0.0001	<0.001*
750 mg - weight	oval	>	round	0.3173	0.3173
	oval	>	oblong	0.0253	0.0337*
	oval	<	mini	0.0389	0.0500
	round	>	oblong	0.2568	0.2889
	round	<	mini	0.0164	0.0227*
	oblong	<	mini	0.0017	0.0040*
1000 mg - weight	oval	<	oblong	0.3173	0.3173
	oval	<	mini	0.0002	<0.001*
	oblong	<	mini	0.0006	0.0020*

Table S5. Results of McNemar test for data of visual perception for younger participants. Given is the *p*-value together with the corrected *p*-value according to Benjamini and Hochberg.

Showcase	Dosage forms (<, >, = indicating trend of swallowability rating)			*p*-value	Corrected *p*-value
oval - shape	125 mg	=	250 mg	1	1
	250 mg	=	500 mg	1	1
	500 mg	>	750 mg	0.3173	0.3427
	750 mg	>	1000 mg	0.0005	0.0015*
	1000 mg	>	1250 mg	<0.0001	<0.001*
round - shape	125 mg	=	250 mg	1	1
	250 mg	>	500 mg	0.3173	0.3427
	500 mg	>	750 mg	0.0003	0.0012*
oblong - shape	125 mg	=	250 mg	1	1
	250 mg	=	500 mg	1	1
	500 mg	>	750 mg	0.3173	0.3427
	750 mg	>	1000 mg	0.0047	0.0115*
	1000 mg	>	1250 mg	<0.0001	<0.001*
capsules	4	=	3	1	1
	3	=	2	1	1
	2	>	1	0.3173	0.3427
	1	>	0	0.0047	0.0115*
	0	>	00	<0.0001	<0.001*
250 mg - weight	oval	=	round	1	1
	oval	=	oblong	1	1
	oval	>	capsule	0.1573	0.2360
	round	=	oblong	1	-1
	round	>	capsule	0.1573	0.2360
	oblong	>	capsule	0.1573	0.2360
500 mg - weight	oval	>	round	0.3173	0.3427
	oval	=	oblong	1	1
	oval	>	capsule	<0.0001	<0.001*
	round	<	oblong	0.3173	0.3427
	round	>	capsule	0.1573	0.2360
	oblong	>	capsule	<0.0001	<0.001*
750 mg - weight	oval	>	round	0.4795	0.4795
	oval	=	oblong	1	1
	oval	<	mini	0.0455	0.0878
	round	<	oblong	0.4795	0.4795
	round	<	mini	0.0143	0.0322*
	oblong	<	mini	0.0455	0.0878
1000 mg - weight	oval	<	oblong	0.2568	0.3427
	oval	<	mini	0.0001	<0.001*
	oblong	<	mini	0.0005	0.0015*

Table S6. Results of Fisher's exact test for comparison of visual perception, data from older and younger participants. Given is the *p*-value, the odds ratio, as well as upper and lower 95% confidence interval boundaries. The given results always implicate a better swallowability rating by younger participants compared to older participants or the same rating for both age categories.

Dosage form	*p*-value	Odds ratio	Lower 95% CI	Upper 95% CI	Corrected *p*-value	Corrected lower CI: 96.5%	Corrected upper CI: 96.5%
125 mg round	#	-	-	-	-	-	-
250 mg round	0.1214	0	-	-	0.1428	-	-
500 mg round	<0.0001	0.04	0.01	0.38	<0.001*	0.00	0.37
750 mg round	<0.0001	0.19	0.08	0.45	<0.001*	0.08	0.48
125 mg oval	#	-	-	-	-	-	-
250 mg oval	#	-	-	-	-	-	-
500 mg oval	<0.0001	0	-	-	<0.001*	-	-
750 mg oval	<0.0001	0.02	0.00	0.15	<0.001*	0.00	0.18
1000 mg oval	<0.0001	0.19	0.08	0.45	<0.001*	0.08	0.48
1250 mg oval	0.0945	0.51	0.21	1.22	0.1181	0.20	1.30
125 mg oblong	#	-	-	-	-	-	-
250 mg oblong	0.5	0	-	-	0.5	-	-
500 mg oblong	0.0001	0	-	-	<0.001*	-	-
750 mg oblong	<0.0001	0.02	0.00	0.14	<0.001*	0.00	0.17
1000 mg oblong	<0.0001	0.09	0.04	0.24	<0.001*	0.03	0.25
1250 mg oblong	0.0146	0.29	0.10	0.83	0.0209*	0.10	0.89
capsule 4	0.2476	0	-	-	0.2606	-	-
capsule 3	0.0589	0	-	-	0.0785	-	-
capsule 2	0.0006	0	-	-	<0.001*	-	-
capsule 1	<0.0001	0.04	0.00	0.29	<0.001*	0.00	0.34
capsule 0	<0.0001	0.13	0.05	0.33	<0.001*	0.05	0.34
capsule 00	0.1395	0.57	0.24	1.34	0.1550	0.22	1.43
750 mg mini	0.0006	0	-	-	<0.001*	-	-
1000 mg mini	0.0001	0	-	-	<0.001*	-	-

Test cannot be calculated.

Table S7. Results of McNemar test to study the influence of visual display on visual perception of different dosage forms. Anticipated swallowability ratings of the same dosage form displayed in the shape- vs weight-showcase were compared. Data are shown for older participants. *p*-values for the individual comparisons are given.

Tablet shape resp. dosage form	Tablet weight resp. capsules size			*p*-value	Corrected *p*-value
	Shape-showcase		Weight-showcase		
oval	250 mg	>	250 mg	0.3173	0.3878
	500 mg	<	500 mg	0.0253	0.0557
	750 mg	<	750 mg	0.0082	0.0451*
	1000 mg	<	1000 mg	0.3657	0.4023
round	250 mg	=	250 mg	1	1
	500 mg	>	500 mg	0.5271	0.5271
	750 mg	<	750 mg	0.0003	0.0033*
oblong	250 mg	>	250 mg	0.1573	0.2884
	500 mg	<	500 mg	0.3173	0.3878
	750 mg	<	750 mg	0.3173	0.3878
	1000 mg	<	1000 mg	0.0209	0.0557
capsules	1	>	1	0.0196	0.0557
	00	>	00	1	1

Table S8. Results of McNemar test to study the influence of visual display on visual perception of different dosage forms. Anticipated swallowability ratings of the same dosage form displayed in the shape- vs weight-showcase were compared. Data are shown for younger participants. *p*-values for the individual comparisons are given.

Tablet shape resp. dosage form	Tablet weight resp. capsules size			*p*-value	Corrected *p*-value
	Shape-showcase		Weight-showcase		
oval	250 mg	=	250 mg	1	1
	500 mg	=	500 mg	1	1
	750 mg	>	750 mg	0.1797	0.4193
	1000 mg	>	1000 mg	0.593	0.6547
round	250 mg	=	250 mg	1	1
	500 mg	=	500 mg	1	1
	750 mg	<	750 mg	0.0114*	0.0798
oblong	250 mg	=	250 mg	1	1
	500 mg	=	500 mg	1	1
	750 mg	>	750 mg	0.0833	0.2916
	1000 mg	>	1000 mg	0.3173	0.5553
capsules	1	>	1	0.5637	0.6547
	00	>	00	0.6547	0.6547

Table S9. Results of McNemar test to study the difference in swallowability after actual deglutition compared to visual perception for the different tablets. Data of visual perception only includes ratings of those participants having actually swallowed the particular tablets. This allows to study significant differences in swallowability ratings for the individual participant. For all comparisons, worse swallowability ratings were anticipated for the visual perception part. Data are shown for older and younger participants. *p*-values for the individual comparisons are given.

Tablet	Older participants		Younger participants	
	p-value	Corrected *p*-value	*p*-value	Corrected *p*-value
250 mg oval	1	1	1	1
500 mg oval	0.4142	0.4142	1	1
750 mg oval	0.0027	0.0041*	0.3173	0.3173
1000 mg oval	0.0003	<0.001*	0.0253	0.0633
250 mg round	1	1	1	1
500 mg round	0.0114	0.0137*	0.3173	0.3173
750 mg round	0.0003	<0.001*	0.0082	0.0410*
1000 mg oblong	0.0003	<0.001*	0.0733	0.1222
